# Mpox Surveillance Based on Rash Characteristics — 13 Emergency Departments, United States, June–December 2023

**DOI:** 10.15585/mmwr.mm7322a1

**Published:** 2024-06-06

**Authors:** Carl T. Berdahl, Anusha Krishnadasan, Kavitha Pathmarajah, Gregory J. Moran, Jesus R. Torres, Matthew Waxman, William Mower, Omai B. Garner, Lorenzo P. Duvergne, Anne W. Rimoin, Pamina M. Gorbach, David A. Talan, EMERGEncy ID NET Study Group, EMERGEncy ID NET Study Group, Brett Faine, Jon K. Femling, James W. Galbraith, Derek Isenberg, Jonathan Jui, Frank LoVecchio, Johanna C. Moore, Utsav Nandi, Richard Rothman, Howard Smithline, Mark T. Steele, Amy M. Stubbs, Sam S. Torbati

**Affiliations:** ^1^Cedars-Sinai Medical Center, Los Angeles, California; ^2^David Geffen School of Medicine at UCLA, Los Angeles, California; ^3^Olive View-UCLA Medical Center, Los Angeles, California; ^4^Ronald Reagan UCLA Medical Center, Los Angeles, California; ^5^UCLA Fielding School of Public Health, Los Angeles, California.; University of Iowa Hospitals & Clinics, Iowa City, Iowa; University of New Mexico Hospitals, Albuquerque, New Mexico; University of Mississippi Medical Center, Jackson, Mississippi; Temple University Hospital, Philadelphia, Pennsylvania; Oregon Health & Science University, Portland, Oregon; Valleywise Health Medical Center, Phoenix, Arizona; Hennepin County Medical Center, Minneapolis, Minnesota; University of Mississippi Medical Center, Jackson, Mississippi; Johns Hopkins Hospital, Baltimore, Maryland; Baystate Medical Center, Springfield, Massachusetts; University Health Truman Medical Center, University of Missouri-Kansas City, Kansas City, Missouri; University Health Truman Medical Center, University of Missouri-Kansas City, Kansas City, Missouri; Cedars-Sinai Medical Center, Los Angeles, California

**Summary**

**What is already known about this topic?**

After the 2022 global mpox outbreak, which primarily affected gay and bisexual men who have sex with men (GBMSM), U.S. cases declined, but low-level transmission continued. Local outbreaks have raised concern about mpox reemergence, including previously unsuspected cases among non-GBMSM.

**What is added by this report?**

During June–December 2023, among 196 patients aged ≥3 months evaluated at 13 U.S. emergency departments for an mpox-compatible rash irrespective of epidemiologic risk factors, three (1.5%) mpox cases were identified, all among unvaccinated GBMSM who had engaged in sex with one or more partners they met through smartphone dating applications.

**What are the implications for public health practice?**

Clinicians should remain vigilant for monkeypox virus infections, particularly among GBMSM, and educate patients about the importance of risk reduction and JYNNEOS vaccination.

## Abstract

In 2022, a global mpox outbreak occurred, primarily affecting gay and bisexual men who have sex with men (GBMSM). To screen for mpox’s reemergence and investigate potentially unsuspected cases among non-GBMSM, prospective surveillance of patients aged ≥3 months with an mpox-compatible rash (vesicular, pustular, ulcerated, or crusted) was conducted at 13 U.S. emergency departments (EDs) during June–December 2023. Demographic, historical, and illness characteristics were collected using questionnaires and electronic health records. Lesions were tested for monkeypox virus using polymerase chain reaction. Among 196 enrolled persons, the median age was 37.5 years (IQR = 21.0–53.5 years); 39 (19.9%) were aged <16 years, and 108 (55.1%) were male. Among all enrollees, 13 (6.6%) were GBMSM. Overall, approximately one half (46.4%) and one quarter (23.5%) of enrolled persons were non-Hispanic White and non-Hispanic Black or African American, respectively, and 38.8% reported Hispanic or Latino (Hispanic) ethnicity. Unstable housing was reported by 21 (10.7%) enrollees, and 24 (12.2%) lacked health insurance. The prevalence of mpox among ED patients evaluated for an mpox-compatible rash was 1.5% (95% CI = 0.3%–4.4%); all persons with a confirmed mpox diagnosis identified as GBMSM and reported being HIV-negative, not being vaccinated against mpox, and having engaged in sex with one or more partners met through smartphone dating applications. No cases were identified among women, children, or unhoused persons. Clinicians should remain vigilant for mpox and educate persons at risk for mpox about modifying behaviors that increase risk and the importance of receiving 2 appropriately spaced doses of JYNNEOS vaccine to prevent mpox.

## Introduction

On May 23, 2022, CDC activated its mpox outbreak response, and on July 23, the World Health Organization declared mpox a Public Health Emergency of International Concern (*1*). Approximately 30,000 U.S. clade II mpox cases were reported in 2022; although cases declined sharply during late 2022, mpox has continued to spread at low levels.*^,^^†^ Whereas the majority of infections occurred among gay and bisexual men who have sex with men (GBMSM) (*1*), cases also occurred among women, children, and other persons with no reported sexual contact, including those experiencing homelessness or working in crowded settings (*2*). Serologic surveys during the peak of the outbreak suggested that some cases went undiagnosed, although the rate of undiagnosed cases among persons at high risk was low (*3*).

Concern regarding mpox resurgence is related to low vaccination coverage among persons at risk for mpox, the possibility of infection in persons who have been vaccinated, and incomplete knowledge about risk factors among persons living in congregate settings (*4*,*5*). Recent reports have described mpox outbreaks in major U.S. metropolitan areas, including Chicago (March–June 2023) (*6*) and Los Angeles (May–August 2023) (*7*).

Emergency departments (EDs) disproportionately care for persons with increased risk, including those susceptible to contracting infectious diseases, sexual and gender minorities, persons living with HIV, and those who are unhoused, work or live in congregate settings, and abuse alcohol or other drugs. Therefore, to screen for mpox’s reemergence among all potentially affected persons, surveillance based on rash characteristics, rather than epidemiologic risk factors or clinician suspicion, was conducted through *EMERGE*ncy ID NET (https://www.emergencyidnet.org), a U.S. ED-based emerging infections surveillance network.

## Methods

### Study Design and Enrollment Qualifications

A multicenter observational mpox surveillance project was conducted at 13 ED hospital sites.^§^ During June–December 2023, patients aged ≥3 months evaluated in a participating ED with an mpox-compatible rash, defined as one or more lesions that appeared pustular, vesicular, crusted, or ulcerated, were enrolled. A qualifying rash was the only entry criterion; epidemiologic mpox risk factors and other illness characteristics were not considered inclusion criteria. Site coordinators received instruction and ongoing feedback regarding rash identification and characterization from project principal and site investigator physicians. Exclusion criteria included the following conditions: 1) previous enrollment, 2) predesignated as not wishing to participate in research, 3) not English- or Spanish-speaking, 4) unable to provide consent, 5) rash present for >4 weeks, and 6) only lesions >2 cm in diameter, excluding erythema.

During the enrollment visit, demographic, historical, and illness characteristics were collected through patient (or parent) and clinician questionnaires; electronic health record review was completed 5–7 days after the enrollment visit. Two skin swabs were collected from lesions located on different body sites (when possible) from each patient. Swabs were tested at UCLA Clinical Microbiology Laboratory by polymerase chain reaction (PCR) that included targets for both nonvariola orthopoxvirus and monkeypox virus.

### Assessment of Sensitivity of Case Finding

To assess case-finding sensitivity and characterize differences between enrolled and eligible nonenrolled patients, project sites performed monthly audits of project-qualifying nonenrolled ED patients and those receiving hospital mpox PCR testing based on the ED provider’s clinical and epidemiologic suspicion during the patient’s usual ED care, which occurred independently of the solely rash-based mpox surveillance project. Audit lists included project-eligible ED patients with rash associated with *International Classification of Diseases, Tenth Revision* (ICD-10) codes R21 (rash and other nonspecific skin eruption), B00 (herpesviral [herpes simplex] infections), B01 (varicella [chickenpox]), B02 (zoster [herpes zoster]), B03 (smallpox), B04 (monkeypox), and B08 (other viral infections characterized by skin and mucous membrane lesions), and with usual-care mpox PCR testing orders. Demographic and illness characteristics of enrolled and eligible nonenrolled patients were compared.

### Data Analysis

Descriptive statistics were used to characterize the study population. The frequency of PCR-diagnosed mpox among ED patients evaluated for an mpox-compatible rash and 95% CIs were calculated using the test of binomial proportion. Data were analyzed using SAS statistical software (version 9.4; SAS Institute). This activity was reviewed by the participating sites’ institutional review boards, deemed not research, and was conducted consistent with applicable federal law.^¶^

## Results

### Enrollee Characteristics

Among 196 enrollees, the median age was 37.5 years (range = 0.7–88 years), 39 (19.9%) were aged <16 years, and 108 (55.1%) were male (Table 1), including 13 (6.6%) GBMSM (Supplementary Table 1, https://stacks.cdc.gov/view/cdc/157004). Approximately one half (91; 46.4%) of enrollees were non-Hispanic White and approximately one quarter (46; 23.5%) were non-Hispanic Black or African American. Hispanic or Latino (Hispanic) ethnicity was reported by 76 (38.8%) enrollees. Twenty-one (10.7%) enrollees reported unstable housing and 24 (12.2%) lacked health insurance.

**TABLE 1:** Demographic and medical history characteristics of patients evaluated for an mpox-compatible rash (N = 196) — 13 emergency departments, United States, June–December 2023

Characteristic	No. (%)*
**Age**	
Median age, yrs (IQR)	37.5 (21.0–53.5)
Median age, range	0.7–88.0
<16 yrs	39 (19.9)
**Sex assigned at birth**	
Female	88 (44.9)
Male	108 (55.1)
**Gender**	
Female	71/157 (45.2)
Male	82/157 (52.2)
Genderqueer/gender nonconforming	1/157 (0.6)
Transgender man/trans man	1/157 (0.6)
Other or declined to answer	2/157 (1.3)
**Sexual orientation**	
Straight or heterosexual	126/157 (80.3)
Lesbian or gay	10/157 (6.4)
Bisexual	4/157 (2.5)
Queer, pansexual, or questioning	3/157 (1.9)
Other	1/157 (0.6)
Don’t know or declined to answer	13/157 (8.3)
**Race and ethnicity**	
Asian, NH	4 (2.0)
Black or African American, NH	46 (23.5)
Native American or American Indian, NH	5 (2.6)
White, NH	91 (46.4)
Hispanic or Latino	76 (38.8)
Multiple races, NH	17 (8.7)
Declined to answer or unable to obtain	7 (3.6)
Other	25 (12.8)
**Insurance status**	
Private	51 (26.0)
Medicaid	74 (37.8)
Medicare	35 (17.9)
Veterans or Tricare	4 (2.0)
Other	20 (10.2)
Not insured	24 (12.2)
Unsure or missing	6 (3.1)
**Unstable housing during the previous 3 months**	21 (10.7)
**Immunocompromised^†^**	24 (12.2)
**Received STI diagnosis in the previous year**	14/157 (8.9)
**HIV-positive (by self-report)**	9/157 (5.7)
**Received mpox vaccine**	2/157 (1.3)
**Sexually active during the previous 3 months**	86/157 (54.8)
**Alcohol and substance use**	
Alcohol binging	39/157 (24.8)
Smoked cigarettes, vaped, or chewed tobacco	51/157 (32.5)
Used cannabis or tetrahydrocannabinol	42/157 (26.8)
Injected drugs	9/157 (5.7)
Noninjection stimulant use	13/157 (8.3)
Noninjection opioid use	11/157 (7.0)
Amyl nitrate (“popper”) use	6/157 (3.8)

**Abbreviations:** NH = non-Hispanic; STI = sexually transmitted infection.

* For questions that were only asked of participants aged ≥16 years (157), the denominator is presented, and percentages are out of 157.

^†^ Defined as currently undergoing treatment for rheumatoid arthritis, HIV/AIDS, or cancer.

### Rash Characteristics

Enrollees had a median of 10 lesions, with a median lesion diameter of 0.5 cm (Table 2). Rashes were described as vesicular (50.5%), crusted (41.8%), pustular (27.0%), and ulcerated (22.5%). Twelve (6.1%) participants were assessed by their treating ED clinician as being likely or very likely to have mpox as the cause of their rash, and 13 enrollees (6.6%) underwent usual-care testing for mpox.

**TABLE 2:** Characteristics of rash and treatment among patients evaluated for an mpox-compatible rash (N = 196) — 13 emergency departments, United States, June–December 2023

Characteristic	No. (%)
Rash lesions*	
Number, median (IQR)	10 (40–20)
Number, range	1–200
Diameter, cm, median (IQR)	0.5 (0.5–1.5)
Diameter, cm, range	0.1–21.0
**Previous visit for current rash**	68 (34.7)
**Days with active rash**	
0–3	69 (35.2)
4–7	65 (33.2)
8–14	43 (21.9)
15–30	19 (9.7)
**Painful rash**	140 (71.4)
**Itchy rash**	135 (68.9)
**Contact with a person who had similar rash**	14 (7.1)
**Reported subjective or measured fever during previous 14 days**	55 (28.1)
**Location of first rash swab**	
Head, face, or neck	34 (17.4)
Trunk	31 (15.8)
Groin or buttocks	25 (12.8)
Upper extremity	44 (22.5)
Lower extremity	39 (19.9)
Oral	22 (11.2)
Anus	1 (0.5)
**Location of second rash swab (n = 190 enrollees)^†^**	
Head, face, or neck	32/190 (16.8)
Trunk	30/190 (15.8)
Groin or buttocks	18/190 (9.5)
Upper extremity	45/190 (23.7)
Lower extremity	46/190 (24.2)
Oropharynx	17/190 (9.0)
Anus	2/190 (1.1)
**Clinician’s suspicion regarding mpox diagnosis**	
Very unlikely	129 (66.5)
Unlikely	45 (23.2)
Neutral	8 (4.1)
Likely	3 (1.6)
Very Likely	9 (4.6)
**Usual-care mpox swab performed**	13 (6.6)
**Usual-care mpox PCR test positive^§^**	2/13 (15.4)
**Surveillance mpox PCR test positive**	3/196 (1.5)
**STI testing results^¶^**	
Chlamydia	4 /18 (22.2)
Gonorrhea	2/14 (14.3)
Herpes	6/25 (24.0)
HIV	3/31 (9.7)
Syphilis	7/25 (28.0)
Trichomonas	2/7 (28.6)
No STI test performed	142 (72.0)
**Medications administered in an ED**	
Antibiotics	42 (21.4)
Antiviral (e.g., acyclovir or valacyclovir)	20 (10.2)
Steroids	19 (9.7)
Tecovirimat (TPOXX)	0 (—)
**ED diagnosis**	
Allergic reaction	3 (1.5)
Cellulitis	16 (8.2)
Contact dermatitis	11 (5.6)
Eczema	7 (3.6)
Hand, foot, and mouth disease	5 (2.6)
Herpes simplex	13 (6.6)
Insect bite	0 (—)
Mpox	3 (1.5)
Rash	59 (30.1)
Scabies	1 (0.5)
Shingles	36 (18.4)
URI, influenza, influenza-like illness, or viral syndrome	0 (—)
Other diagnosis	105 (53.6)
**ED disposition**	
Discharged home	153 (78.1)
Discharged to SNF	2 (1.0)
Discharged to self-care (street/unhoused)	3 (1.5)
Admitted to this hospital	35 (17.9)
Left against medical advice	3 (1.5)
**Medications prescribed at ED discharge**	
Antibiotics	49 (25.0)
Antiviral (e.g., acyclovir or valacyclovir)	46 (23.5)
Steroids	33 (16.8)
Tecovirimat (TPOXX)	1 (0.5)
**45-day follow-up phone call completed (n = 131)**	131 (66.8)
**Rash status at 45 days**	
Resolved	89/131 (67.9)
Better	30/131 (22.9)
About the same	9/131 (6.9)
Worse	3/131 (2.3)

**Abbreviations:** ED = emergency department; HPV = human papillomavirus; PCR = polymerase chain reaction; SNF = skilled nursing facility; STI = sexually transmitted infection; URI = upper respiratory infection.

* Three participants had lesion counts noted as “too numerous to count” and were not included in this calculation. Lesion counts were missing for four participants.

^†^ Second rash swab was not obtained from six participants.

^§^ Two patients receiving testing through the surveillance project were also suspected through their usual ED care of having mpox and received hospital mpox PCR testing. Both patients received positive mpox test results by the surveillance and hospital laboratory tests.

^¶^ Number with positive test result among total number tested.

### Mpox Patient Characteristics

Among all 196 enrollees, three (1.5%) received a positive monkeypox virus PCR test result; all three identified as GBMSM and reported being HIV-negative, not vaccinated against mpox, and having engaged in sex with one or more partners they met through smartphone dating applications (Table 3). All three patients were assessed by the treating ED clinician as being “very likely” to have mpox. No mpox cases were identified among women, children, or persons experiencing homelessness.

**TABLE 3:** Characteristics of enrollees with positive monkeypox virus test results — California, Minnesota, and Oregon, June–December 2023

Characteristic	Patient 1	Patient 2	Patient 3
Study site location	Los Angeles, California	Minneapolis, Minnesota	Portland, Oregon
Age, yrs	29	30	42
Race and ethnicity	Black or African American, NH	White, NH	Declined race, Hispanic or Latino
Location of lesions	Groin and oropharynx	Face, neck, and abdomen	Hand and genitals
No. of lesions	Three	Three	Two
Duration of rash at ED evaluation	6 days	Approximately 2 weeks	10 days
Patient description of lesions	Painful	Tender and itchy	Painful and itchy
Clinician description of lesions, lesion diameter	Pustular, crusted, 0.5–1 cm	Crusted, 2 cm	Vesicular, 0.5 cm
Additional signs and symptoms	Fever, chills, myalgias, fatigue, headache, sore throat, and diarrhea	Chills, myalgias, fatigue, nasal congestion, lymphadenopathy, diarrhea, tenesmus, and dysuria	Fever, chills, myalgia, fatigue, headache, lymphadenopathy, and dysuria
Sexual orientation	Gay	Gay	Gay
Previous evaluation and findings	STI clinic 3 days earlier, positive mpox test result, and presumptive syphilis diagnosis	Different ED examination 13 days earlier, and provisional diagnosis of Klebsiella, mpox or MRSA (pending mpox test result)	Previously examined in urgent or primary care where he was told he might have mpox
Treatment before ED visit	Prescribed tecovirimat and underwent treatment for suspected syphilis with penicillin G benzathine	Prescribed trimethoprim-sulfamethoxazole	Prescribed doxycycline, azithromycin, and valacyclovir
Social and sexual behavior during previous 3 months	Sexually active, including with male partners met via smartphone apps, and inconsistent condom use	Sexually active, including with male partners met via smartphone apps, participated in oral and anal sex, and never used condoms; used a noninjectable stimulant; attended at least one large, crowded gathering (e.g., music festival, rave, or other crowded social event); participated in group sex and sex parties; and traded sex for money, drugs, a place to stay, and gifts	Sexually active, including with male partners met via smartphone apps, used condoms consistently, and reported opioid use and amyl nitrate use
Living situation	Unstable housing (currently living with roommate)	Stable housing with one roommate	Stable housing, living with two roommates
STI	HIV-negative and taking HIV preexposure prophylaxis	Received diagnosis of and treatment for chlamydia and gonorrhea in the previous year, HIV-negative, and not taking HIV preexposure prophylaxis	HIV-negative and taking HIV preexposure prophylaxis
Mpox vaccination	No	No	No
ED disposition	Admitted for IV hydration and continued tecovirimat treatment with dehydration due to oropharyngeal lesions	Discharged from an ED with mpox diagnosis and no discharge prescriptions	Discharged from an ED with diagnosis of possible mpox and bacteremia, and a discharge prescription for amoxicillin

**Abbreviations:** ED = emergency department; IV = intravenous; MRSA = methicillin-resistant *Staphylococcus aureus*; NH = non-Hispanic; STI = sexually transmitted infection.

### Comparison of Enrolled and Eligible Nonenrolled Participants

A total of 67 patients received testing for mpox at hospital laboratories at study sites as part of their usual ED care (13 of whom were also enrolled and tested through the project); three (4.5%) received positive test results, two of whom were also identified in the study. Among all 196 enrolled participants, 13 (6.6%) also received usual-care testing, two of whom received a positive hospital PCR test result, which was concordant with the project test results (Supplementary Table 2, https://stacks.cdc.gov/view/cdc/157005) (Table 2). Among 991 nonenrolled patients with qualifying rash associated with ICD-10 codes, 54 (5.5%) received usual-care testing for mpox, one (1.9%) of whom received a positive result. This patient, a male aged 25 years, was not included because he was examined in an ED during a period outside of project staff member coverage hours; no further demographic or risk information was available. Overall, the enrolled population was demographically similar to the eligible nonenrolled audited patients, but enrolled patients were more likely than were nonenrolled patients to have been admitted to a hospital (17.9% versus 9.9%).

## Discussion

During June–December 2023, the prevalence of mpox among patients in 13 U.S. EDs who were evaluated for an mpox-compatible rash was low: among 196 enrolled patients, three (1.5%) received a positive monkeypox virus PCR test result, all of whom were unvaccinated GBMSM who engaged in sexual activity with partners they met through smartphone applications. Only an estimated 23% of the U.S. population at risk for mpox exposure had received vaccination during May 2022–January 2023 (*8*). No cases were identified among women, children, and unhoused persons. These findings add to a body of evidence indicating that mpox continues to circulate among persons at risk for mpox, primarily GBMSM with sexual risk factors (*4*,*6*,*7*), and underscore the importance of educating persons at risk for mpox regarding behavioral risks and encouraging these persons to be vaccinated (*9*).

### Limitations

The findings in this report are subject to at least four limitations. First, the project was limited to 13 EDs and included a small sample size; thus, these findings might not be generalizable to other areas. Second, case-finding sensitivity was suboptimal because site staff members were unable to enroll all eligible patients for reasons that included the lack of night and weekend project personnel coverage and rapid discharge of eligible patients. However, the representativeness of the project population was supported by the audit, which indicated that enrolled and nonenrolled eligible patients were demographically similar and included a similar proportion of persons for whom hospital mpox testing was ordered by clinicians as part of their usual ED care. Further, approximately three times as many project patients received mpox testing (196) as did ED patients who received usual ED care (67), which identified only one additional case. Despite the presence of an mpox-compatible rash, a clinician’s index of suspicion for mpox likely was lower, and testing was infrequently ordered for non-GBMSM patients. Third, eligibility based on rash features might have been inconsistent across sites because of variation in staff member interpretation of rash descriptors. To mitigate this limitation, site coordinators attended a series of onboarding meetings and subsequent monthly meetings to address ongoing questions about rash appearances. Finally, the sample size did not permit investigation of factors associated with mpox, such as the actual number of sex partners, knowingly engaging in sex with a person with an mpox-compatible rash, or frequency of nonsexual skin-to-skin contact; future work with larger sample sizes and more cases could facilitate assessment of these risk factors.

### Implications for Public Health Practice

Mpox cases continue to occur in the United States. In addition, mpox remains endemic in other parts of the world. Although no clade I cases have yet been reported in the United States (*10*), public health officials are currently closely monitoring clade I in the Democratic Republic of the Congo because it appears to be more transmissible and to result in more severe disease than does clade II, which caused the 2022 global outbreak. Clinicians should remain vigilant for monkeypox virus infections, particularly among GBMSM at increased risk, and educate patients on ways to lower their risk, including the importance of receiving 2 appropriately spaced doses of JYNNEOS vaccine to prevent mpox (*9*).

## Footnotes

*
https://www.cdc.gov/poxvirus/mpox/response/2022/index.html

†
https://worldhealthorg.shinyapps.io/mpx_global/

§ Baystate Medical Center (Springfield, Massachusetts); Cedars-Sinai Medical Center (Los Angeles, California); Hennepin County Medical Center (Minneapolis, Minnesota); Johns Hopkins Hospital (Baltimore, Maryland); Olive View-UCLA Medical Center (Los Angeles, California); Oregon Health and Science University (Portland, Oregon); Ronald Reagan UCLA Medical Center (Los Angeles, California), Temple University Hospital (Philadelphia, Pennsylvania); University Health Truman Medical Center, University of Missouri-Kansas City (Kansas City, Missouri); University of Iowa Hospitals and Clinics (Iowa City, Iowa); University of Mississippi Medical Center (Jackson, Mississippi); University of New Mexico Hospitals (Albuquerque, New Mexico); and Valleywise Health Medical Center (Phoenix, Arizona).

¶ 45 C.F.R. part 46.102(l)(2), 21 C.F.R. part 56; 42 U.S.C. Sect. 241(d); 5 U.S.C. Sect. 552a; 44 U.S.C. Sect. 3501 et seq.

## References

[1] McQuiston JH, Braden CR, Bowen MD, The CDC domestic mpox response—United States, 2022–2023. MMWR Morb Mortal Wkly Rep 2023;72:547–52. 10.15585/mmwr.mm7220a2 37200231 PMC10205168

[2] Sharpe JD, Charniga K, Byrd KM, Possible exposures among mpox patients without reported male-to-male sexual contact—six U.S. jurisdictions, November 1–December 14, 2022. MMWR Morb Mortal Wkly Rep 2023;72:944–8. 10.15585/mmwr.mm7235a2 37651279

[3] Minhaj FS, Singh V, Cohen SE, Prevalence of undiagnosed monkeypox virus infections during global mpox outbreak, United States, June–September 2022. Emerg Infect Dis 2023;29:2307–14. 10.3201/eid2911.230940 37832516 PMC10617324

[4] Hazra A, Zucker J, Bell E, ; SHARE-NET writing group. Mpox in people with past infection or a complete vaccination course: a global case series. Lancet Infect Dis 2024;24:57–64. 10.1016/S1473-3099(23)00492-9 37678309

[5] Sachdeva H, Shahin R, Ota S, Preparing for mpox resurgence: surveillance lessons from outbreaks in Toronto, Canada. J Infect Dis 2024;229(Suppl 2):S305–12. 10.1093/infdis/jiad533 38035826 PMC10965211

[6] Faherty EAG, Holly T, Ogale YP, Notes from the field: emergence of an mpox cluster primarily affecting persons previously vaccinated against mpox—Chicago, Illinois, March 18–June 12, 2023. MMWR Morb Mortal Wkly Rep 2023;72:696–8. 10.15585/mmwr.mm7225a6 37347713 PMC10328474

[7] Leonard CM, Poortinga K, Nguyen E, Mpox outbreak—Los Angeles County, California, May 4–August 17, 2023. MMWR Morb Mortal Wkly Rep 2024;73:44–8. 10.15585/mmwr.mm7302a4 38236779 PMC10803096

[8] Owens LE, Currie DW, Kramarow EA, JYNNEOS vaccination coverage among persons at risk for mpox—United States, May 22, 2022–January 31, 2023. MMWR Morb Mortal Wkly Rep 2023;72:342–7. 10.15585/mmwr.mm7213a4 36995962 PMC10078841

[9] CDC. Mpox vaccine recommendations. Atlanta, GA: US Department of Health and Human Services, CDC; 2024. Accessed May 31, 2024. https://www.cdc.gov/poxvirus/mpox/vaccines/vaccine-recommendations.html

[10] Kibungu EM, Vakaniaki EH, Kinganda-Lusamaki E, ; International Mpox Research Consortium. Clade I–associated mpox cases associated with sexual contact, the Democratic Republic of the Congo. Emerg Infect Dis 2024;30:172–6. 10.3201/eid3001.231164 38019211 PMC10756366

